# Microstructure Evolution at Ni/Fe Interface in Dissimilar Metal Weld between Ferritic Steel and Austenitic Stainless Steel

**DOI:** 10.3390/ma16186294

**Published:** 2023-09-20

**Authors:** Xiaogang Li, Junfeng Nie, Xin Wang, Kejian Li, Haiquan Zhang

**Affiliations:** 1 Institute of Nuclear and New Energy Technology, Key Laboratory of Advanced Reactor Engineering and Safety of Ministry of Education, Tsinghua University, Beijing 100084, China; wxx@tsinghua.edu.cn (X.W.); haiquanzh@tsinghua.edu.cn (H.Z.); 2 Department of Mechanical Engineering, Tsinghua University, Beijing 100084, China; kejianli@mail.tsinghua.edu.cn

**Keywords:** dissimilar metal weld, Ni/Fe interface, phase transformation, martensitic structures, interfacial performance

## Abstract

The formation and evolution of microstructures at the Ni/Fe interface in dissimilar metal weld (DMW) between ferritic steel and austenitic stainless steel were investigated. Layered martensitic structures were noted at the nickel-based weld metal/12Cr2MoWVTiB steel interface after welding and post-weld heat treatment (PWHT). The formation of the interfacial martensite layer during welding was clarified and its evolution during PWHT was discussed by means of scanning electron microscopy (SEM), electron backscatter diffraction (EBSD), electron probe microanalysis (EPMA), focused ion beam (FIB), transmission electron microscopy (TEM), energy dispersive X-ray (EDX), transmission kikuchi diffraction (TKD), phase diagrams, and theoretical analysis. In as-welded DMW, the Ni/Fe interface structures consisted of the BCC quenched martensite layer and the FCC partially mixed zone (PMZ), which was the result of inhomogeneous solid phase transformation due to the chemical composition gradient. During the PWHT process, the BCC interfacial microstructure further evolved to a double-layered structure of tempered martensite and quenched martensite newly formed by local re-austenitization and austenite–martensite transformation. These types of martensitic structures induced inhomogeneous hardness distribution near the Ni/Fe interface, aggravating the mismatch of interfacial mechanical properties, which was a potential factor contributing to the degradation and failure of DMW.

## 1. Introduction

In coal-fired and nuclear power plants, ferritic heat-resistant steels are widely employed to manufacture tubes, and they are always used with austenitic stainless steels (ASSs) or nickel-based alloys to form dissimilar metal welds (DMWs) [1,2,3]. In DMWs, ASSs and nickel-based alloys with excellent creep rupture strength and good oxidation resistance serve in higher temperature positions and ferritic steels serve in lower temperature positions [1,2,3]. Welding is, therefore, an important technical means of fabricating components for power plants [4]. There are thousands of DMWs in one power plant [2]. DMWs will be used in more high-temperature applications in the future. Ultra supercritical power plants are expected to operate at a steam temperature of 760 °C, in which the tube serving at 760 °C in the superheater is made of nickel-based alloy and the tube serving below 650 °C is made of ferritic steel; thus, DMWs are employed [5]. In high-temperature gas-cooled reactors, the superheater section is made of 800H alloy while the evaporator section is made of low-alloy ferritic steel, and there are DMWs between them. In addition, DMWs are also widely used in welding rotors [6], reheaters [7], steam tubes [8], and other high-temperature applications [9] in power plants. For the above DMWs, the austenitic steel or nickel-based alloy side is generally reliable, and DMW service life is mainly affected by the ferritic steel side with relatively poor performance [10]. Austenitic filler metals were used in early DMWs [11]. However, there would be high thermal stress at the interface between the austenitic weld metal (WM) and ferritic base metal (BM) during DMW service in high-temperature environments due to the huge difference in the coefficient of thermal expansion (CTE) between the two [12]. Meanwhile, serious carbon migration could occur from the BM across the interface to the WM during high-temperature service. As a result, DMWs fabricated with austenitic filler metal always fractured along the WM/BM interface, resulting in premature failure [12]. In recent years, nickel-based filler material has been widely used to replace austenitic filler material, which has greatly improved the service performance of DMWs [11,13,14]. For example, the service life of 2.25Cr-1Mo/316 DMW fabricated with nickel-based filler metal was five times that of DMW fabricated with austenitic filler metal, [15] which was due to the reduction of the CTE difference between the two sides of the WM/ferritic BM interface and weakened carbon migration. Therefore, the Ni/Fe interface between nickel-based WM and ferritic BM is the key factor to improving the performance of this type of DMW used in power plants. However, DMW with nickel-based WM still cannot be guaranteed to be safe in service for 200,000 h without failure, and its typical failure time at 600 °C is about 70,000 h [16], which is closely related to special microstructures at the Ni/Fe interface [17,18]. The Fe/Ni interface microstructure after welding seriously affects the evolution and performance of the interface during long-term service, but it is rarely paid attention in detail [19]. Consequentially, the formation and evolution of interfacial microstructures before service is a prerequisite of failure analysis of DMWs.

The formation of microstructures at the Ni/Fe interface is determined by dissimilar materials and the welding method. During the welding heating process, nickel-based filler material is molten and a small amount of ferritic BM is also molten. Therefore, there is a partially mixed zone (PMZ) [2] at the edge of the welding pool, in which the molten filler material and molten BM are unevenly mixed. After the welding cooling process, the Ni/Fe interface is formed between the PMZ and unmolten BM. There is a steep chemical composition gradient in the interior of the PMZ and a composition interface between the PMZ and unmolten BM, which results in complex microstructure evolution near the Ni/Fe interface. In addition, the microstructures near the Fe/Ni interface might evolve further during the post-weld heat treatment (PWHT) process [20]. Special microstructures were observed near the Ni/Fe interface in early years, i.e., the martensite layer [21,22] and interfacial precipitates [3,13,16,23]. In recent years, the accuracy of the experimental equipment for characterization has improved, which provides possibilities for further clarifying microstructure evolution. In addition to experimental observations, theoretical explanation is also necessary for Ni/Fe interface microstructure evolution. Therefore, characterization of the microstructures at the Ni/Fe interface and evolution analysis are urgently needed to clarify premature failure of DMWs and are important aspects of interface metallurgy. In particular, the formation and evolution of the interfacial microstructures are related to the chemical composition, crystallographic relationship, and temperature. Thus, the obtained results of microstructure characterization require validation using detailed chemical composition, orientation relationship, and phase diagram analyses.

This work investigated the microstructures near the Ni/Fe interface in DMW between ASS and ferritic steel with nickel-based filler material by means of scanning electron microscopy (SEM), electron backscatter diffraction (EBSD), transmission electron microscopy (TEM), transmission kikuchi diffraction (TKD), energy dispersive X-ray (EDX), and electron probe microanalysis (EPMA). The mechanisms of interfacial microstructure formation and evolution during welding and PWHT were discussed with the aid of phase diagram and orientation analyses. In addition, nanoindentation testing was performed in different micro-regions near the Ni/Fe interface to preliminarily evaluate the performance of interface structures.

## 2. Experimental

### 2.1. Materials and DMW Fabrication

The BMs used for DMW fabrication were 12Cr2MoWVTiB ferritic steel and ASS. The two BMs were tubes with 60 mm external diameter and 7 mm wall thickness. The above size is a common specification for steam tubes in power plants. The chemical composition (wt. %) of 12Cr2MoWVTiB steel was 1.9 Cr, 0.12 C, 0.64 Mn, 0.55 Mo, 0.42 W, 0.40 V, 0.54 Si, 0.005 B, 0.12 Cu, and Fe balance. The chemical composition (wt. %) of ASS was 18.4 Cr, 0.07 C, 11.3 Ni, 1.52 Mn, 0.75 Nb, 0.60 Si, and Fe balance.

The tungsten inert gas (TIG) welding method was used for manufacturing the DMW between 12Cr2MoWVTiB steel tube and ASS tube. The groove angle was 60° and multi-layer welding was applied. The filler material was Inconel 82 (ERNiCr-3) wire with 2.4 mm diameter. The chemical composition (wt. %) of ERNiCr-3 was 18.0–22.0 Cr, 0.10 C, 3.0 Fe, 2.5–3.5 Mn, 1.0–3.0 Nb, ≤0.75 Ti, ≤0.5 Cu, ≤0.12 Co, 0.5 Si, and ≥67.0 Ni balance. The welding was performed under a current of 100–130 A, a voltage of 8–12 V, and welding speed of 25–50 mm/s. The shielding gas was pure argon at a flow rate of 9–12 L/min. To reduce the welding stress, BMs were preheated at 200 °C before welding, and the interlayer temperature was controlled at 300 °C during welding.

After welding, PWHT was conducted to release residual stress in the DMW, according to the standard BS-2633-1987. The heating rate of PWHT was related to the diameter and wall thickness of the tube, which was defined as 200 °C/h. The holding temperature and holding time were set to 700 °C and 1 h, respectively. The cooling rate was related to the diameter and wall thickness of the tube, which was defined as 250 °C/h.

### 2.2. Characterization Methods for Microstructure

In this study, the microstructure was characterized by optical microscopy (OM), SEM, and EBSD, and the chemical composition was determined by EDX and EPMA. Meanwhile, nanoindentation testing was performed to evaluate the mechanical properties of the microstructures of interest. According to the different characterization requirements, the samples were prepared using different methods. The samples were cut from the tube and contained the whole DMW, with the size of 25 mm × 5 mm × 2 mm. The samples for microstructure characterization were ground to 5000 grit, and then they were mechanically polished to a 2.5 µm surface finish. In this study, microstructure evolution at the interface between nickel-based WM and ferritic BM was the main focus; thus, the 12Cr2MoWVTiB steel side was chemically etched with a solution consisting of ethyl alcohol (97 vol%) and nitric acid (3 vol%). The interfacial microstructures were examined by OM and SEM. To further investigate the interfacial microstructures, EBSD was employed to obtain the crystal information and EDX was employed to obtain the chemical composition information. The sample for EBSD examination was mechanically polished and then prepared using ion beam polishing by an ion beam milling system. Ion beam polishing was carried out ten times at 2~6 kV voltage and 1.3~2.5 mA gun current. The angle between the gun and the polishing surface was 3°. To obtain the chemical composition profile, point by point EPMA quantitation was performed across the WM/ferritic steel interface. For further nanoscale analysis, a focused ion beam (FIB) was used to accurately extract interfacial samples for TEM and TKD observations. EDX and selected area electron diffraction (SAED) were performed in the TEM system.

To evaluate the performance of different microstructures near the Ni/Fe interface, a nanoindentation system (FT-I04, FemtoTools, Buchs, Switzerland) was used to obtain the hardness in different subregions near the Ni/Fe interface. The sample for the nanoindentation test was prepared by mechanical and ion beam polishing.

## 3. Results

### 3.1. Microstructure of the 12Cr2MoWVTiB Steel Matrix

**Figure 1:**
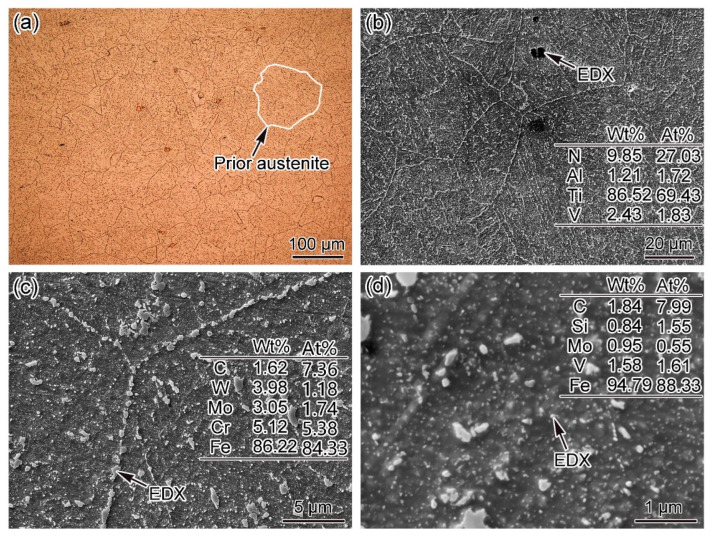
Microstructures of 12Cr2MoWVTiB steel: (**a**) OM image; (**b**) SEM image; (**c**) microscale carbides; (**d**) nanoscale carbides.

In this study, the ferritic BM for DMW fabrication was low-alloy bainitic steel, which was developed in China. The microstructures of 12Cr2MoWVTiB steel were examined by OM and SEM, as shown in Figure 1. Figure 1a shows an OM image of the 12Cr2MoWVTiB matrix. The prior austenite was equiaxed grain and the grain size was about 100 μm. The SEM image in Figure 1b clearly shows the morphologies of the 12Cr2MoWVTiB matrix. The as-received 12Cr2MoWVTiB steel had been tempered, and thus its matrix was tempered bainite, which had a lath-shaped morphology decorated by many precipitated phases. The Ti-rich nitrides acted as nucleation positions in the casting process. There were many microscale precipitated phases dotted at prior austenite boundaries, which were Cr-rich carbides detected by EDX, as shown in Figure 1c. There were many nanoscale precipitated phases in the interior of grains, which were V-rich carbides detected by EDX, as shown in Figure 1d. The above carbides played a role in precipitation strengthening and improved deformation resistance at high temperature [24,25].

### 3.2. Layered Structure at the WM/12Cr2MoWVTiB Steel Interface Formed by Welding

**Figure 2:**
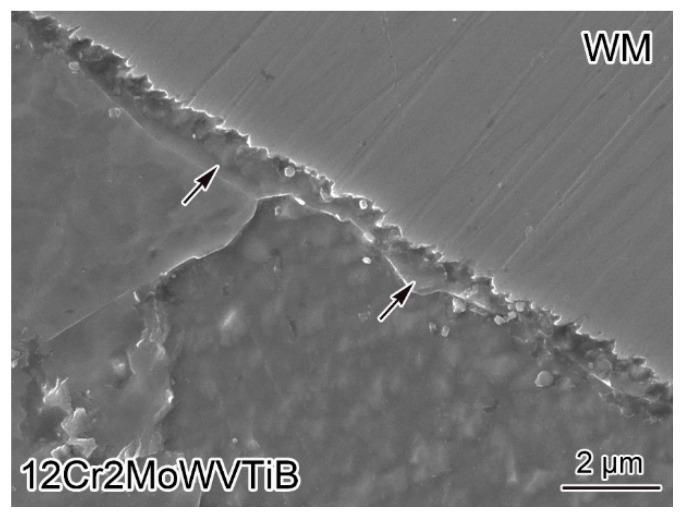
SEM image of the Ni/Fe interface between nickel-based WM and 12Cr2MoWVTiB steel after welding.

To reveal the whole evolution process of the interfacial microstructures, characterization of the interface structures after welding is essential. The Cr content in nickel-based WM was ~20% and that in 12Cr2MoWVTiB steel was ~2%. Using the solution containing nitric acid to corrode the DMW, the 12Cr2MoWVTiB steel was chemically etched, while the WM was not, and thus the Ni/Fe interface was displayed. Figure 2 clearly shows the interface between WM and 12Cr2MoWVTiB steel. During the welding heating process, the carbides in the heat-affected zone near the interface had been dissolved and thus could not be observed after welding, which is a common behavior in HAZ due to the welding heat cycle [26]. It is worth noting that there was a layer along the interface, as marked by the black arrows in Figure 2. For the as-welded DWM, no substructures or carbides were observed in the interior of the layer. In the literature [21], a layer with substructures at the interface was observed by OM, but the layer in this study was not completely consistent with this report. The type of layer in this study needed to be further determined.

**Figure 3:**
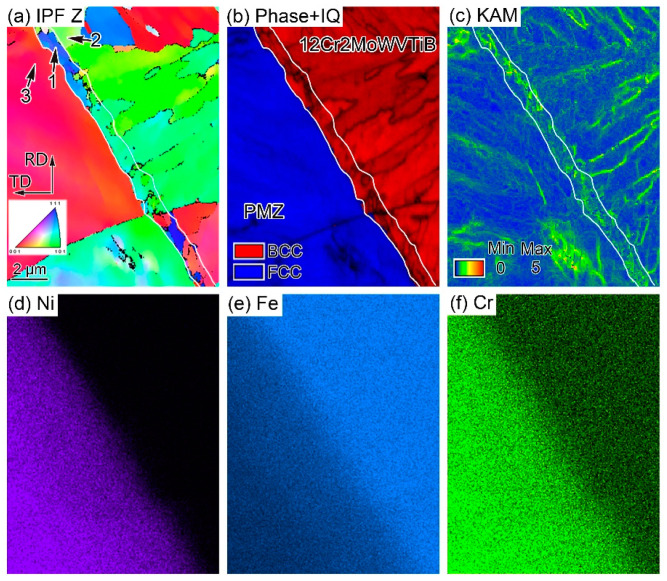
Crystal structure and chemical composition of the layer at the Ni/Fe interface in the DMW after welding: (**a**) inverse pole figure (IPF) Z map, (**b**) phase + image quality (IQ) map, (**c**) kernel average misorientation (KAM) map, obtained by EBSD with 40 nm step size; (**d**) Ni elemental map, (**e**) Fe elemental map, (**f**) Cr elemental map, obtained by EDX and corresponding to the location in (**a**).

The Ni/Fe interface between WM and 12Cr2MoWVTiB steel was investigated by EBSD and EDX, as shown in Figure 3. The orientation of the layered structure (see position 1 in Figure 3a) was different from that of the adjacent BM (see position 2 in Figure 3a) and adjacent WM (see position 3 in Figure 3a). The layer at the interface had a body-centered cubic (BCC) structure (see phase + IQ map in Figure 3b). The KAM map obtained by EBSD could semi-quantitatively reflect the dislocation density distribution. The dislocation density of the layer mentioned above was higher than that of the 12Cr2MoWVTiB matrix, as shown in the KAM map in Figure 3c. The scanning electron microscope used contained both EBSD and EDX detectors, and thus the composition distribution in the EBSD detection location in Figure 3 could be simultaneously obtained, as shown in Figure 3d–f. The PMZ between WM and 12Cr2MoWVTiB steel showed a gradient change in Ni, Cr, and Fe contents. In the PMZ, the Ni and Cr contents gradually increased and the Fe content gradually decreased from BM to WM.

Furthermore, the chemical compositions of the interfacial microstructures were quantitatively determined by EDX, and the results are listed in Table 1. The Cr content of the layer at the interface was 5.85% (wt. %), which was higher than the nominal Cr content of 12Cr2MoWVTiB steel. This indicated that the layer was not formed by solidification of molten BM. Meanwhile, the contents of Ni and Fe in the layer were 13.95% (wt. %) and 79.29% (wt. %), respectively, which indicated that the layer was formed by the mixing of molten BM and molten filler metal. No Ni element was detected at position 2 in Figure 3a, the Cr content was only 2.99% (wt. %), and the Fe content was 95.61% (wt. %), which indicated this position was in the 12Cr2MoWVTiB matrix. The Ni content at position 3 in Figure 3a increased to 33.36% (wt. %), the Cr content to 9.92% (wt. %) and the Fe content to 55.76% (wt. %), indicating that position 3 was in the PMZ. According to the above results, the layer at the interface was located at the edge of the PMZ and adjacent to BM, which meant that its formation process might be related to the PMZ.

**Figure 4:**
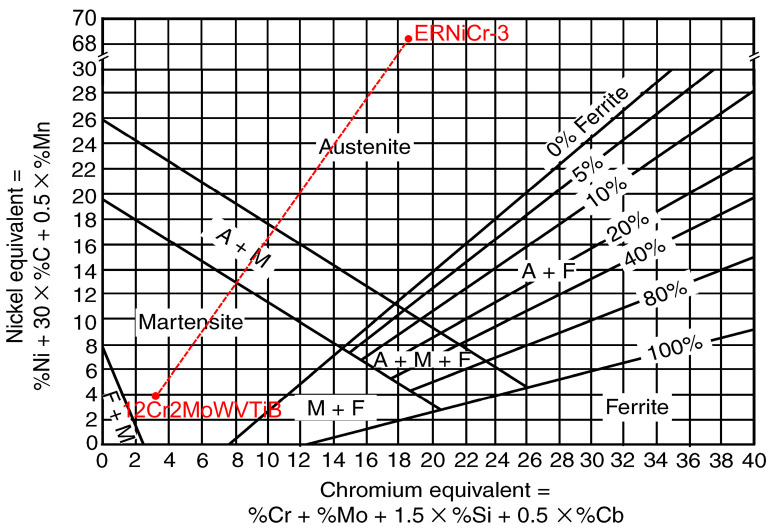
Interfacial microstructure prediction by Schaeffler diagram. The coordinates of the red points in the figure were determined by the chemical compositions of ferritic BM and nickel-based WM. The red dotted line in the figure represents the change in Cr_eq_ and Ni_eq_ from BM to WM.

To clarify the type of layer at the interface, a Schaeffler diagram was used for reference. A Schaeffler diagram can be used to preliminarily predict the microstructure in welds of alloy steels and stainless steels by means of arc welding. The chemical compositions of WM and ferritic BM were converted into chromium equivalents (Cr_eq_) and nickel equivalents (Ni_eq_) according to Equations (1) and (2), marked in the Schaeffler diagram as the horizontal and vertical axes, as shown in the red points in Figure 4. The chemical composition of the PMZ transformed from that of BM to that of WM. The dotted line connecting the two red points in Figure 4 expressed the change in Cr_eq_ and Ni_eq_ in the PMZ. The dotted line could only traverse through martensitic and austenitic regions, but not through ferritic regions. Therefore, the possibility that the BCC layer at the interface after welding was ferrite could be excluded. Meanwhile, it could be inferred that the BCC layer was martensite formed in the PMZ.

(1) $${\mathrm{Cr}}_{\mathrm{eq}}=\%\mathrm{Cr}+\%\mathrm{Mo}+1.5\times \%\mathrm{Si}+0.5\times \%\mathrm{Cb}$$

(2) $${\mathrm{Ni}}_{\mathrm{eq}}=\%\mathrm{Ni}+30\times \%C+0.5\times \%\mathrm{Mn}$$

### 3.3. Microstructures at the WM/12Cr2MoWVTiB Steel Interface after PWHT

Toughness is a critical indicator for steam tubes in power plants [27,28], so PWHT should be performed on tubes containing DMWs to improve their toughness and release residual stress [29,30]. Meanwhile, the chemical composition gradient in the PMZ is large, and there are regions in a thermodynamically unstable state. Microstructure evolution is likely to occur during PWHT. The Ni/Fe interface that undergoes PWHT is actually the initial state before service. Therefore, it is necessary to characterize the interfacial microstructures after PWHT.

**Figure 5:**
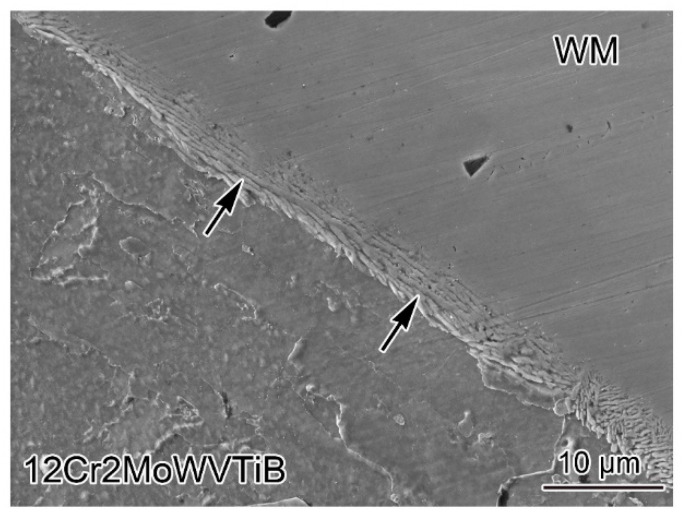
SEM image of the Ni/Fe interface between nickel-based WM and 12Cr2MoWVTiB steel in the DMW after PWHT.

12Cr2MoWVTiB BM in the DMW after PWHT was etched by the solution containing nitric acid, and then the Ni/Fe interface between WM and BM was observed. Figure 5 shows the microstructures near the interface. There was still a layered microstructure at the interface, as marked by the black arrows in Figure 5. It is worth noting that lath-shaped substructures were present in the layered microstructure in the DMW after PWHT, which was different from the layer (see Figure 2) in the as-welded DMW. The results of morphological observation implied that the layered microstructure in the DMW after PWHT seemed to be tempered martensite.

**Figure 6:**
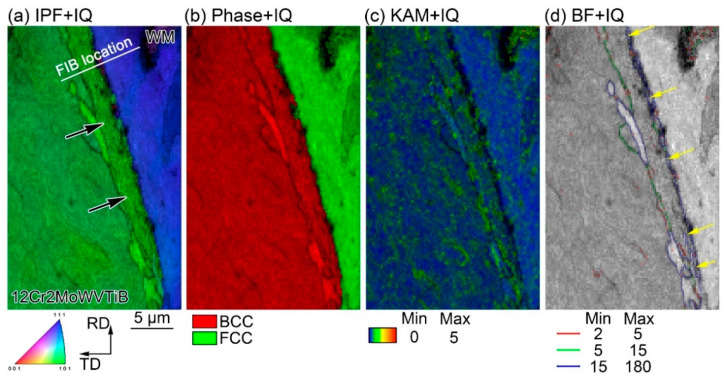
Crystal structure of the layered microstructure at the Ni/Fe interface in the DMW after PWHT, obtained by EBSD with 80 nm step size: (**a**) IPF + IQ map; (**b**) phase + IQ map; (**c**) KAM + IQ map; (**d**) boundary figure (BF) + IQ map.

In addition, EBSD was performed on the Ni/Fe interface in the DMW after PWHT, as shown in Figure 6. There was still a layered structure between 12Cr2MoWVTiB BM and WM, as marked by the black arrows in Figure 6a. The layered microstructure had a BCC structure (see phase + IQ map in Figure 6b). Meanwhile, the KAM map was used to semi-quantitatively reveal the dislocation density distribution, as shown in Figure 6c. The dislocation density of the layered microstructure was still high. Specifically, the BF + IQ map showed a band of finer structures with large angle grain boundaries in the layered microstructure connected to the WM, as indicated by the yellow arrows in Figure 6d. These finer microstructures were newly formed during the PWHT process, not the welding process. A martensite layer with a width of 1 to 3 μm along the WM/ferritic steel interface was reported in a DMW after PWHT in the literature [2], but the finer microstructures similar to those shown in Figure 6d was not mentioned.

**Figure 7:**
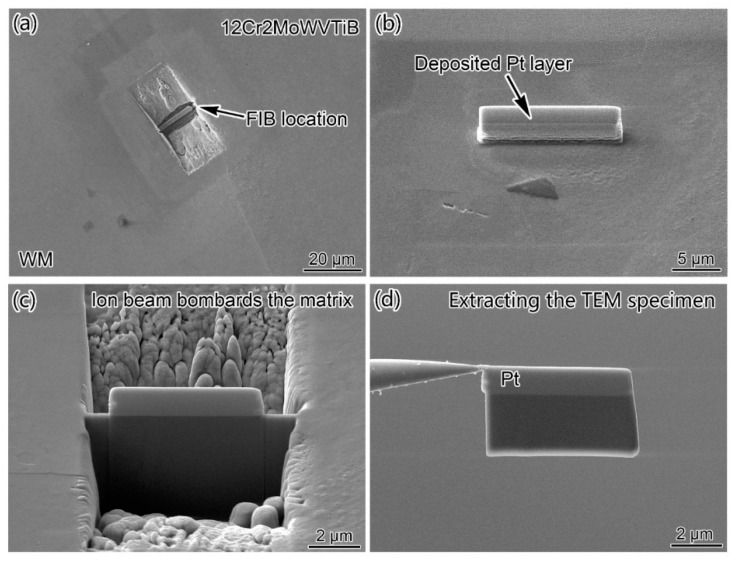
Process of preparing TEM sample by FIB: (**a**) sampling location; (**b**) depositing Pt on sampling location; (**c**) ion beam bombardment matrix; (**d**) extracting the sample.

In addition to the layered microstructure, finer structures appeared along the interface after PWHT. Thus, it was necessary to characterize the interfacial microstructures and chemical composition in more detail. FIB was employed to accurately extract the interfacial sample in the DMW after PWHT for TEM observation. The position marked by the white line in Figure 6a indicates the FIB location. Figure 7 shows the preparation process for the FIB sample. Since the interfacial regions contained three different microstructures, namely 12Cr2MoWVTiB steel, layered microstructure, and nickel-based WM, the hardness gradient made the thinning process difficult during preparation of the TEM sample. Therefore, only part of the final sample prepared by FIB could be used for TEM observation.

**Figure 8:**
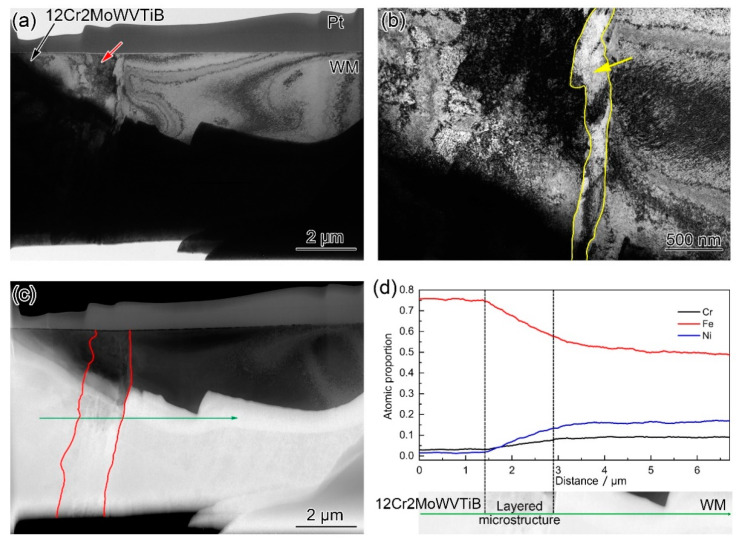
TEM and EDX results for the microstructures at the Ni/Fe interface in the DMW after PWHT: (**a**) bright-field TEM image; (**b**) detailed bright-field TEM image in a region of interest in (**a**); (**c**) high-angle annular dark-field (HAADF) image corresponding to (**a**); (**d**) EDX line-scanning across the layered microstructures, as indicated by the green line in (**c**).

Figure 8 shows the TEM and EDX results for the interfacial microstructures. Figure 8a clearly displays the layered structure with a width of ~1 μm between 12Cr2MoWVTiB steel and WM (marked by the red arrow in Figure 8a). It is worth noting that there was a band of bright white microstructures with a width of ~200 nm on the right side of the layered microstructure, as marked by the yellow arrow in Figure 8b. K. Y. Shin et al. [31] conducted TEM observational analysis of the nickel-based WM/P92 interface, but did not note the above ~200 nm microstructure. This new microstructure had not been reported, so further analysis was warranted. Figure 8c shows the HAADF image corresponding to Figure 8a, and the layered structure along the interface could also be clearly observed, as outlined by the red line. Figure 8d shows the EDX line-scanning results across the interface, which revealed the chemical composition change. There was a chemical composition gradient inside the layered microstructures.

**Figure 9:**
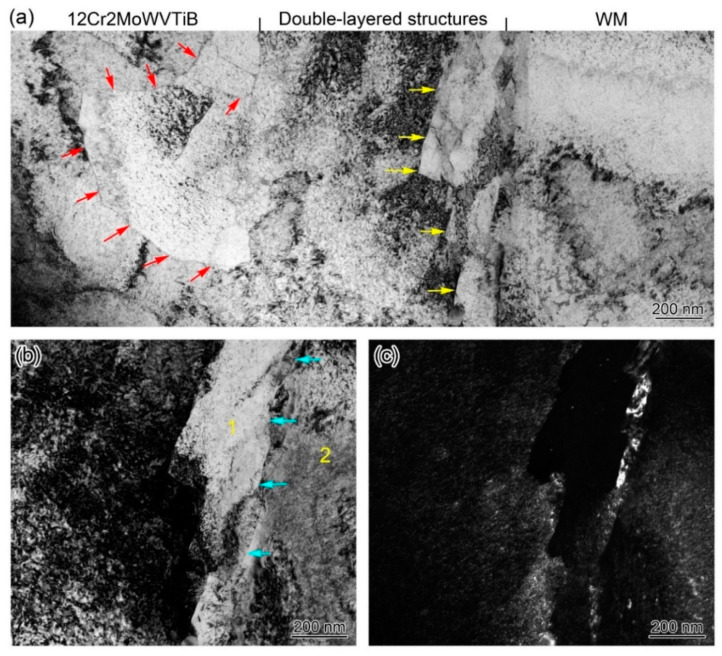
Morphology of interfacial microstructures in the DMW after PWHT: (**a**) Bright-field TEM image; (**b**) detailed bright-field TEM image; (**c**) detailed dark-field TEM image.

Figure 9 shows the detailed results of the TEM observation of the interfacial microstructures in the DMW after PWHT. The equiaxed grains in the 12Cr2MoWVTiB matrix could be clearly observed on the left side of the layered microstructure, and the red arrows marked in Figure 9a indicate their grain boundaries. Furthermore, the band of finer structures adjacent to WM was clearly displayed. There was a boundary between the layered microstructure and the finer microstructures, as indicated by the yellow arrows in Figure 9a. Detailed bright-field and dark-field TEM images in a region of interest in Figure 9a are shown in Figure 9b,c. The above morphological results by TEM observation suggested the layered microstructure and the finer microstructures were not the same type.

**Figure 10:**
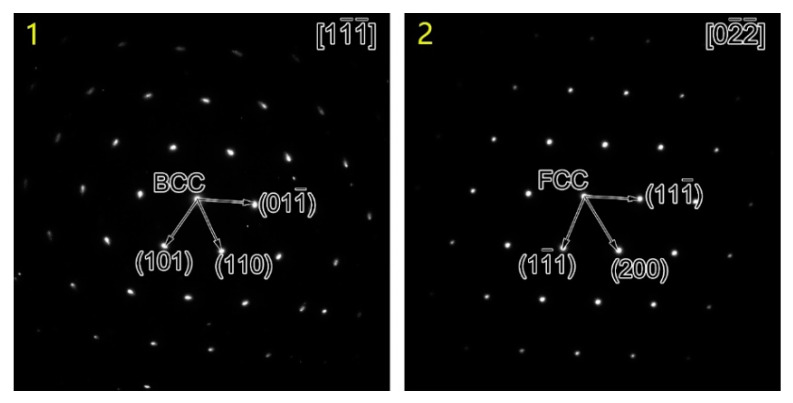
Selected area electron diffraction (SAED) results for positions 1 and 2 in Figure 9c.

In addition, SAED was performed on the band of finer microstructures (position 1 in Figure 9b) and its adjacent microstructure (position 2 in Figure 9b), and the results are shown in Figure 10. The band of finer microstructures (position 1 in Figure 9b) was BCC and its adjacent microstructure (position 2 in Figure 9b) was FCC, which indicated that the microstructure on the right side of the band of finer microstructures was already WM. There was a clear boundary between the band of finer microstructures and WM, as marked by the blue arrows in Figure 9b. Figure 9c shows a dark-field TEM image corresponding to Figure 9b. Importantly, there were no substructures in the interior of the band of finer microstructures. The band of finer microstructures observed by TEM corresponded to the finer structures along the interface observed by EBSD (marked by the yellow arrows in Figure 6d). According to the experimental results, it could be speculated that the band of finer microstructures with a width of ~200 nm was likely to be quenched martensite newly formed in the PWHT process.

## 4. Discussion

### 4.1. Solidification and Phase Transformation during Welding

To investigate the microstructure evolution at the Ni/Fe interface in the DMW, the formation of the layer during welding (see the microstructure marked by the black arrows in Figure 2) needed to be clarified first. Thus, the solidification and phase transformation of the interfacial microstructures were discussed. The layer at the interface was part of the PMZ and located at the edge of the PMZ. The formation process of microstructures in the PMZ could be defined from the phase diagram. Since the main metallic elements in the PMZ were Fe, Cr, and Ni, the microstructure formation could be predicted by the Fe-Cr-Ni phase diagram.

**Figure 11:**
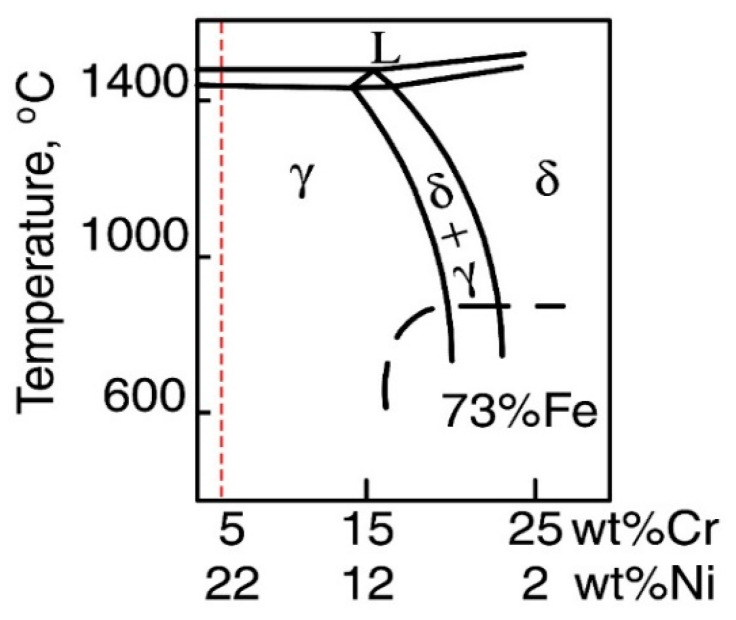
Fe-Cr-Ni pseudo-binary phase diagram (73% Fe) [33].

**Figure 12:**
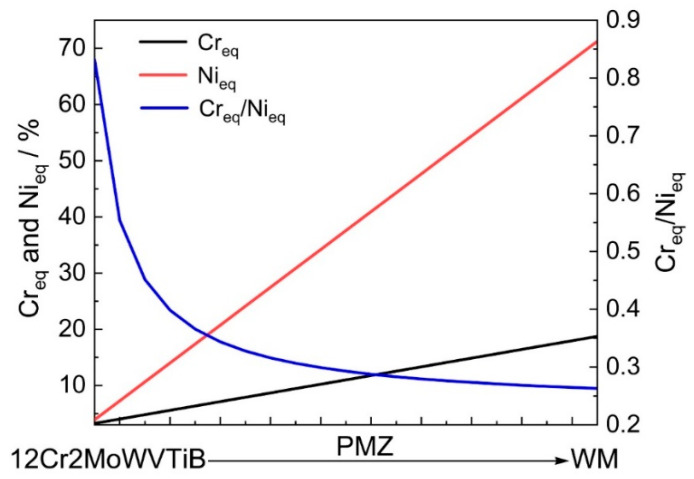
Change trends of Cr_eq_ and Ni_eq_ in the PMZ between 12Cr2MoWVTiB BM and nickel-based WM.

First, the solidification process in the PMZ was considered. According to the Fe content in the layer at the interface, the pseudo-binary phase diagram at 73% Fe was selected to analyze the solidification process, as shown in Figure 11. Based on the different chemical compositions, there were four paths of solidification for the Fe-Cr-Ni alloy, which were as follows: Mode A, L→L + A→A; Mode AF, L→L + A→L + A + (A + F)_eutectic_→A + F_eutectic_; Mode FA, L→L + F→L + F + (F + A)_peritectic/eutectic_→F + A; Mode F, L→L + F→F→F + A. With the increase in Cr_eq_/Ni_eq_, solidification path changes along modes A, AF, FA, and F resulted in increased δ ferrite content in matrix [32]. However, according to the Schaeffler diagram in Figure 4, ferrite could not be formed in the PMZ in this study. According to the results of the chemical composition of position 1 in Table 1, Cr_eq_ and Ni_eq_ of the layer at the interface after welding were 5.85% and 14.41%, respectively. The Cr_eq_/Ni_eq_ of the layer was 0.41, as marked by the red dashed line in Figure 11. Therefore, the solidification path at this position was mode A. It could be confirmed that δ ferrite would not form in the PMZ. The chemical compositions of 12Cr2MoWVTiB steel and filler metal were taken as the endpoints of the chemical composition of the PMZ, and the contents of each element made a linear transition in the PMZ. According to the above simplification, the change trends of Cr_eq_ and Ni_eq_ in the PMZ were obtained, as shown by the black and red lines in Figure 12. Cr_eq_/Ni_eq_ is shown by the blue line in Figure 12. From the BM side to the WM side, Cr_eq_/Ni_eq_ in the PMZ gradually decreased, and thus the other parts in the PMZ, except the layer, were located to the left of the red dotted line in Figure 11. Therefore, the solidification path of the whole PMZ was L→L + A→A, and the microstructures in the PMZ were austenite after solidification and before solid phase transformation.

**Figure 13:**
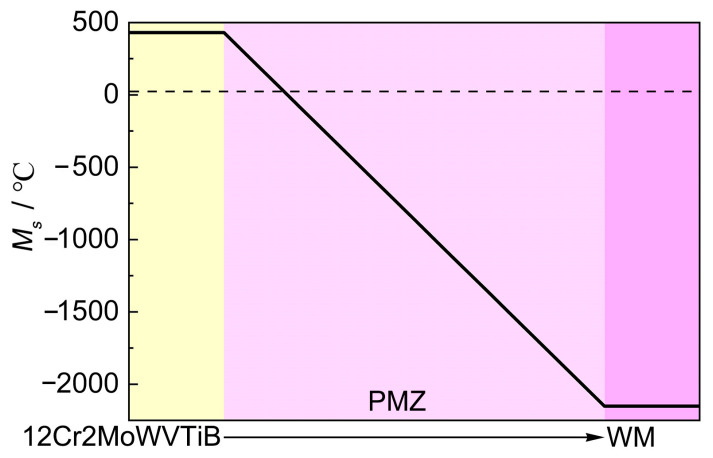
Change trend of *M*_s_ in the PMZ between 12Cr2MoWVTiB BM and nickel-based WM.

Furthermore, the solid phase transformation in the PMZ was analyzed. The chemical composition in the PMZ gradually changed from BM to WM, and thus the transformation of austenite in the PMZ after solidification to martensite could be considered during the subsequent cooling process. The chemical compositions of 12Cr2MoWVTiB steel and filler metal were taken as the endpoints of the chemical composition of the PMZ and the contents of each element made a linear transition. Then, *M*_s_ in the PMZ was calculated according to Equation (3) and the result is shown in Figure 13. From the BM side to the WM side, *M*_s_ in the PMZ rapidly decreased to below room temperature, but theoretically there would be a narrow region adjacent to BM where *M*_s_ was above room temperature, as shown in Figure 13. This meant that austenite in this region could transform into martensite during cooling to room temperature. Combining the experimental results in Section 3.2 with the above theoretical analysis indicated that the layer at the interface after welding was martensite.

(3) $$M_{s}=540-(479C+6.3Mn+36.3Ni+10.8Cr+46.6Mo)$$

**Figure 14:**
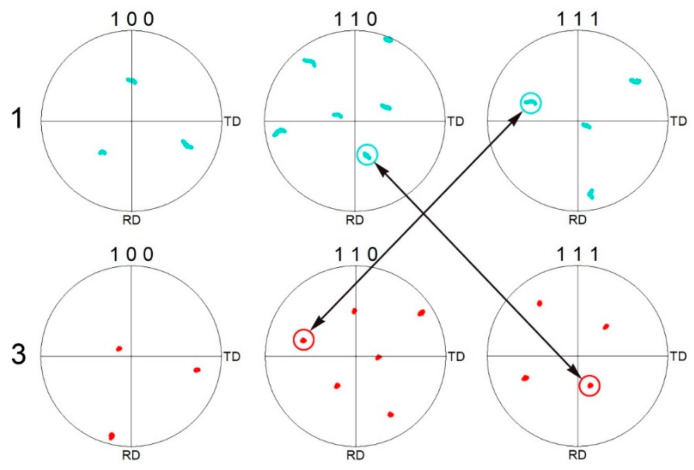
Analysis of orientations of the layer (position 1 in Figure 3a) and its adjacent PMZ (position 3 in Figure 3a) by pole figure (PF) maps.

In addition, the orientation relationship between the martensite layer at the interface (position 1 in Figure 3a) and its adjacent FCC PMZ (position 3 in Figure 3a) was revealed by PF maps using EBSD technology, as shown in Figure 14. The martensite layer after welding and its adjacent FCC PMZ followed the K–S relationship [34,35]. This meant that during the cooling process, when position 1 was austenite, position 3 was also austenite. Positions 1 and 3 belonged to the same prior austenite grain. The high Ni content in position 3 improved austenite stability, and the microstructure at position 3 was still austenite after cooling to room temperature. The low Ni content in position 1 was insufficient to preserve the austenite to room temperature; thus, the austenite transformed into interfacial martensite. The above viewpoint was proven by the experimental evidence that positions 1 and 3 maintained the K–S relationship. The solidification and solid phase transformation in the PMZ during welding were clarified.

### 4.2. Evolution of Interfacial Microstructure during PWHT

In the DMW after PWHT, there were lath-shaped substructures in the interfacial martensite (see Figure 5), which were different from the layer at the interface after welding (see Figure 2). The literature [2] claimed that carbon atoms and carbides near the interface might be redistributed during PWHT, but the crystal structure and other chemical elements remained unchanged. However, in the DMW after PWHT, advanced characterization revealed that there was a band of finer microstructures adjacent to WM (see Figure 6d and Figure 9), which seemed to be quenched martensite based on the morphological characteristics. This implied that solid phase transformation occurred at the Ni/Fe interface during the PWHT process.

The chemical composition of the martensite layer at the Ni/Fe interface after PWHT was measured by EPMA, and the results are shown in Table 2. It should be pointed out that the width of the interfacial martensite layer of the EPMA-detected site was about 3 μm, while the detected diameter by EPMA was about 1 μm. Thus, the EPMA results in Table 2 cannot represent the chemical composition of the entire interfacial martensite layer and reflect the change in the chemical composition of its interior. It is worth noting that there was 9.26% Ni (wt. %) in the interfacial martensite layer, and the Ni element could greatly reduce *A*_c1_. Based on the chemical composition results in Table 2, *A*_c1_ was calculated to be 447 °C according to Equation (4), which indicated that austenitization might occur in some regions of the interfacial martensite layer during PWHT at 700 °C. Meanwhile, by substituting the chemical composition results in Table 2 into Equation (3), *M*_s_ was calculated to be 81 °C, which was higher than room temperature. This meant that the austenite newly formed in the interfacial martensite layer during PWHT might be transformed into quenched martensite again during cooling.

(4) $$A_{c1}=723-25Mn-30Ni+25Si+25Mo$$

**Figure 15:**
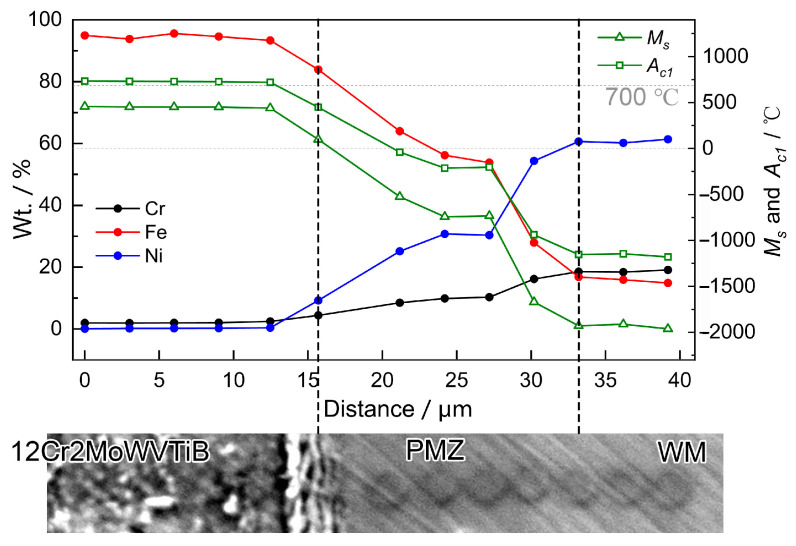
Chemical composition profile across the Ni/Fe interface in the DMW after PWHT, obtained by EPMA.

Furthermore, point-by-point EPMA was performed to obtain the chemical composition change across the interface, as shown in Figure 15. The spacing between two EPMA points was 3 μm. The EPMA quantitative results were used to calculate *A*_c1_ and *M*_s_. From the 12Cr2MoWVTiB steel side to the WM side, *A*_c1_ and *M*_s_ decreased rapidly in the PMZ due to the increased Ni content. The chemical composition of the interfacial martensite layer transitioned continuously from that of 12Cr2MoWVTiB steel to that of FCC PMZ. *A*_c1_ of the interfacial martensite layer adjacent to 12Cr2MoWVTiB steel was higher than 700 °C, which indicated that austenization would not occur in the above microstructure during the PWHT process. *A*_c1_ of the interfacial martensite layer adjacent to FCC PMZ was obviously lower than 700 °C and *M*_s_ was also above room temperature, which meant that the microstructures in this region would be re-austenized during the PWHT process and could be transformed into quenched martensite during the subsequent cooling process. Therefore, finer quenched martensite was newly formed in the region adjacent to FCC PMZ after PWHT.

**Figure 16:**
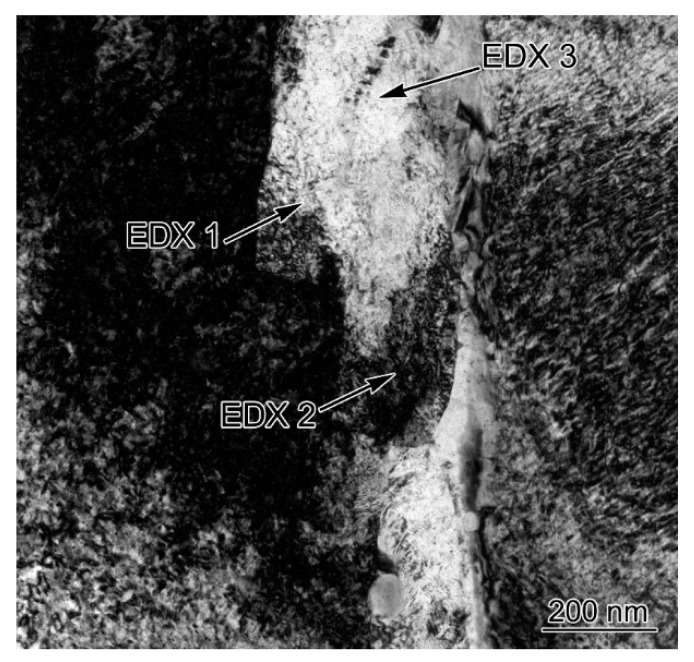
The quenched martensite layer at the Ni/Fe interface in the DMW after PWHT.

The width of the finer quenched martensitic layer formed after PWHT was only about 200 nm. The chemical composition of the finer quenched martensitic layer was further analyzed using EDX in the TEM system. The detection locations (EDX 1, EDX 2, and EDX 3) are marked in Figure 16, and the results are shown in Table 3. The Cr and Ni contents (wt. %) in the above three random detection locations were ~4% and ~10%, respectively. *A*_c1_ and *M*_s_ of the finer quenched martensitic layer were calculated using Equations (3) and (4), as shown in Table 3. *A*_c1_ of the detection locations was ~400 °C. Meanwhile, *M*_s_ of the detection locations was higher than room temperature. Thus, the narrow region adjacent to FCC PMZ was experimentally confirmed to be re-austenized and transformed finer quenched martensite during PWHT at 700 °C. These results indicated that the part at the Ni/Fe interface that had not be re-austenized would transform into tempered martensite. It should be noted that there was inevitable deviation in the chemical composition determined by EDX in the TEM system, and the EDX results only assisted in providing evidence for the conclusions of the previous theoretical analysis.

**Figure 17:**
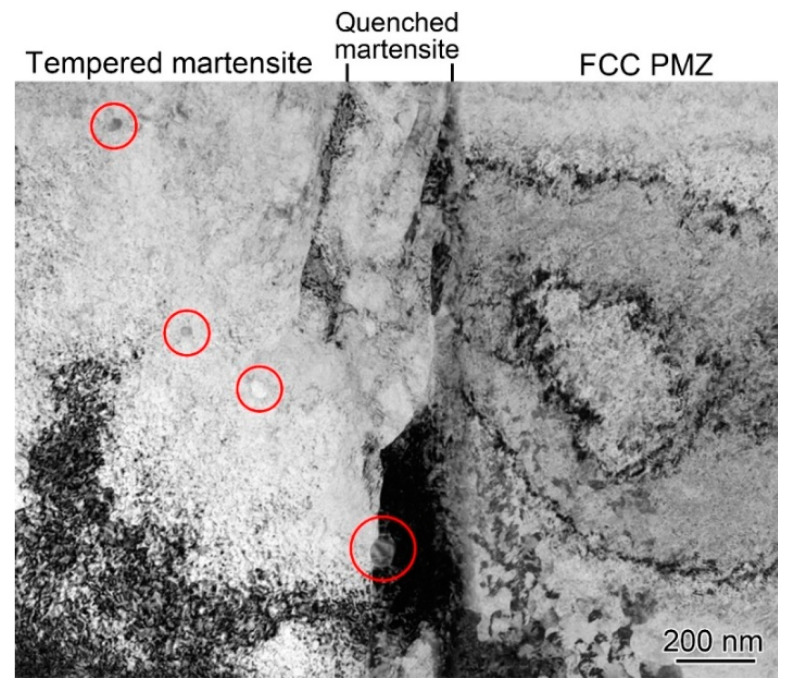
Carbides in the interior of the interfacial martensite layer of the DMW after PWHT.

In addition, carbides were formed at the Ni/Fe interface after PWHT, as shown in Figure 17. A few carbides were observed in the interior of the tempered martensite layer and at the interface between the tempered and quenched martensite, as marked by red circles in Figure 17. The interfacial carbides did not grow and coarsen during PWHT, and their average size was only 40 nm, which was related to the short time of the PWHT.

**Figure 18:**
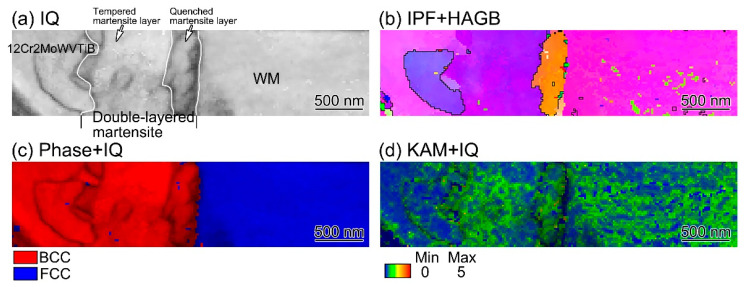
TKD results at the Ni/Fe interface in the DMW after PWHT: (**a**) IQ map; (**b**) IPF + high angle grain boundary (HAGB) map; (**c**) phase + IQ map; (**d**) KAM + IQ map. The step size for TKD was 20 nm.

SAED in the TEM system could only provide crystal information in a limited area. In order to confirm the double-layered structures of quenched and tempered martensite at the Ni/Fe interface after PWHT, TKD was performed on the TEM sample to investigate the interfacial microstructures. The width of the quenched martensite layer was about 200 nm. In order to obtain exact results, the step size of TKD was selected as 20 nm. The results are shown in Figure 18. The IQ map in Figure 18a shows the wider tempered martensite layer and the finer quenched martensite layer. The IPF + HAGB map in Figure 18b clearly shows two kinds of martensite at the Ni/Fe interface. The phase + IQ map in Figure 18c reveals the double-layered BCC structure of the interfacial martensite and the FCC structure of WM. There was high dislocation density in the finer interfacial microstructure, as shown in Figure 18d, which also indicated that the finer interfacial microstructure was quenched martensite. The experimental results of the crystal structures in a large area confirmed the microstructure evolution during the PWHT process.

### 4.3. Inhomogeneous Mechanical Properties near the Ni/Fe Interface

**Figure 19:**
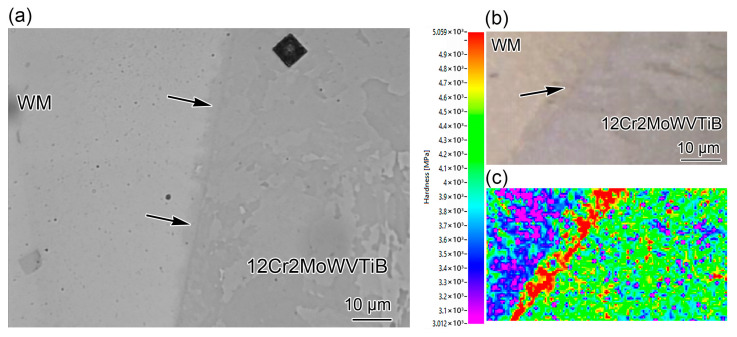
Inhomogeneity of microstructure and hardness of the Ni/Fe interface between nickel-based WM and 12Cr2MoWVTiB steel after PWHT: (**a**) OM image of the sample prepared by ion beam polishing; (**b**) rested area of nanoindentation; (**c**) hardness distribution map.

Compared with the nickel-based WM and austenitic stainless steel BM side, the ferritic steel side with relatively poor properties is considered the weakest position. In particular, fracture along the Ni/Fe interface between WM and ferritic steel is the most common and dangerous form of premature failure in engineering, which leads to a much lower service life of the DMW than of ferritic BM [11,36,37,38,39,40]. When failure occurs along the Ni/Fe interface, the DMW might fail within half to a third of its design life [16]. Thus, the mechanical properties of the Ni/Fe interface are important to the reliability of the whole DMW. However, the interfacial martensite layer was narrow and it was difficult to evaluate the mechanical properties of the complex interface containing different microstructures by conventional test methods. Thus, nanoindentation was employed to clarify the mechanical properties of different microstructures near the Ni/Fe interface in the DMW after PWHT. Researchers have used nanoindentation to analyze the hardness across the fusion line and found that there was no significant change in the hardness near the interface [41]. However, the nanoindentation traces reported above were obtained at points across the interface with a point spacing of 3 μm, which was not likely to detect interface structures with a width of ~1 μm along the interface. This limited the analysis of interfacial properties. Thus, in this study, the hardness of complex structures near the interface was evaluated using an array test of nanoindentation with small point spacing, using a high-resolution nanoindentation system and special sample preparation, and the results are shown in Figure 19. Nanoindentation testing is sensitive to the sample surface. Before the nanoindentation test, the sample was prepared by mechanical and ion beam polishing, as shown in Figure 19a. The microstructure marked by the black arrows in Figure 19a is the interfacial martensite layer. An array of nanoindentation was performed in the area of 78 μm × 48 μm, which ensured that different interfacial microstructures were included in the tested area. The indentation depth was maintained at 95 nm to accurately measure the hardness of the microstructures at the interface. The load during the nanoindentation testing was ~980 uN. The spacing between indentation points was at least ten times the indentation depth, so that the two indentation points did not affect each other and the indentation results were effective. Thus, the spacing between indentation points was set as 1 μm, and the holding time was 2 s. The OM image in Figure 19b is the detected area of nanoindentation, in which the interfacial martensite layer is marked by the black arrow. A total of 3781 indentation points were obtained, and the hardness distribution near the Ni/Fe interface is shown in Figure 19c. The nanoindentation results showed that the hardness of the interfacial martensite was obviously higher than that of the PMZ on the left and that of 12Cr2MoWVTiB steel on the right. The higher hardness might be attributed to the substructures, carbides, and high dislocation density in the interior of the interfacial martensite. When the DMW was subjected to external load, the mismatch of hardness near the Ni/Fe interface could lead to uncoordinated deformation and thus would aggravate the stress concentration at the Ni/Fe interface. Under the combined effect of the different CTE and creep strength of both sides of the interface, the Ni/Fe interface would be susceptible to failure by accelerated creep deformation. Thus, it can be speculated that the interfacial martensite layer would contribute to premature failure.

## 5. Conclusions

The microstructural formation and evolution at the nickel-based WM/ferritic steel interface in DMW were investigated and discussed using multi-scale characterization technologies and phase transformation analysis. Based on the results, the evolution mechanisms of Ni/Fe interface structures during welding and PWHT were revealed. The main conclusions are as follows:

(1) Heterogenous interface structures were found along the Ni/Fe interface between nickel-based WM and ferritic steel, consisting of a martensitic layer with a BCC structure and a PMZ with a FCC structure. A K–S relationship existed between the BCC martensitic layer and the FCC PMZ.
(2) During the welding process, the solidification path of the whole PMZ was L→L + A→A. There was a large Ni content gradient in the PMZ, which led to inhomogeneous solid phase transformation during cooling. The region adjacent to ferritic steel in the PMZ contained a lower Ni content, resulting in a higher *M*_s_ point, and a BCC quenched martensite layer was formed after welding. Due to the high Ni content in the region adjacent to WM in the PMZ, the austenite was maintained after cooling, and the FCC PMZ was formed after welding.
(3) During the PWHT process, the BCC quenched martensite layer would further evolve, in which the region adjacent to the FCC PMZ was re-austenized and then finer quenched martensite was newly formed after cooling. Meanwhile, the quenched martensite in the region adjacent to ferritic steel was transformed into tempered martensite with a few carbides formed inside.
(4) The heterogenous microstructure near the Ni/Fe interface aggravated the mismatch of interfacial mechanical properties. Due to the presence of substructures, carbides, and high dislocation density, the hardness of the interfacial martensite was obviously higher than that of the two sides, which might be an adverse factor that deteriorates the performance of DMWs.

## Figures and Tables

**Table 1:** Chemical composition of positions 1~3 in Figure 3a (wt. %).

Position	Cr	Ni	Mn	C	Si	Fe
1	5.85	13.95	0.91	—	—	79.29
2	2.99	—	0.74	0.98	0.67	94.63
3	9.92	33.36	—	0.96	—	55.76

**Table 2:** Chemical composition of interfacial martensite after PWHT (wt. %), obtained by EPMA.

C	Cr	Ni	Mn	Mo	Si	Nb	Co	Cu	Fe
0.11	4.42	9.26	0.68	0.39	0.37	0.0093	0.11	0.034	Balance

**Table 3:** Chemical composition of interfacial quenched martensite newly formed in the PWHT process (wt. %), obtained by EDX in the TEM system.

	Cr	Ni	Mn	Si	Fe	*A*_c1_	*M*_s_
1	4.29	9.79	0.88	0.29	Balance	415	130
2	4.23	9.89	0.94	0.24	Balance	409	126
3	4.41	10.39	0.98	0.25	Balance	393	106

## Data Availability

Not applicable.
